# Inhibition of ANGPT2 activates autophagy during hypertrophic scar formation via PI3K/AKT/mTOR pathway^⋆^

**DOI:** 10.1016/j.abd.2021.12.005

**Published:** 2022-10-19

**Authors:** Hongxin Chen, Kai Xu, Chao Sun, Si Gui, Juanjuan Wu, Song Wang

**Affiliations:** a School of Medicine, Wuhan University of Science and Technology, Wuhan, Hubei, China; b Department of Burn and Plastic Surgery, General Hospital of Central Theater Command of People’s Liberation Army, Wuhan, Hubei, China; c Hubei Key Laboratory of Central Nervous System Tumor and Intervention, Wuhan, Hubei, China; d The Sixth Resignation Cadre Sanatorium of Shandong Province Military Region, Qingdao, China

**Keywords:** Autophagy, Angiopoietin-2, Cell proliferation, Cicatrix, hypertrophic, Class II phosphatidylinositol 3-kinases, Extracellular matrix, Fibroblast

## Abstract

**Background:**

Hypertrophic scar (HS), a fibroproliferative disorder caused by aberrant wound healing following skin injuries such as burns, lacerations and surgery, is characterized by invasive proliferation of fibroblasts and excessive extracellular matrix (ECM) accumulation. The dysregulation of autophagy is the pathological basis of HS formation. Previously, angiopoietin-2 (ANGPT2) was found to be overexpressed in HS fibroblasts (HSFs) compared with normal skin fibroblasts. However, whether ANGPT2 participates in the process of HS formation and the potential molecular mechanisms are not clear.

**Objective:**

This study is intended to figure out the role of ANGPT2 and ANGPT2-mediated autophagy during the development of HS.

**Methods:**

RT-qPCR was used to detect ANGPT2 expression in HS tissues and HSFs. HSFs were transfected with sh-ANGPT2 to knock down ANGPT2 expression and then treated with MHT1485, the mTOR agonist. The effects of sh-ANGPT2 or MHT1485 on the proliferation, migration, autophagy and ECM accumulation of HSFs were evaluated by CCK-8 assay, Transwell assay and western blotting. The expression of PI3K/Akt/mTOR pathway-related molecules (p-PI3K, p-Akt and p-mTOR) was assessed by western blotting.

**Results:**

ANGPT2 expression was markedly upregulated in HS tissues and HSFs. ANGPT2 knockdown decreased the expression of p-PI3K, p-Akt and p-mTOR. ANGPT2 knockdown activated autophagy and inhibited the proliferation, migration, and ECM accumulation of HSFs. Additionally, the treatment of MHT1485, the mTOR agonist, on ANGPT2-downregulated HSFs, partially reversed the influence of ANGPT2 knockdown on HSFs.

**Study limitations:**

The study lacks the establishment of more stable *in vivo* animal models of HS for investigating the effects of ANGPT2 on HS formation in experimental animals.

**Conclusions:**

ANGPT2 downregulation represses growth, migration, and ECM accumulation of HSFs via autophagy activation by suppressing the PI3K/Akt/mTOR pathway. Our study provides a novel potential therapeutic target for HS.

## Introduction

Hypertrophic Scar (HS), a fibroproliferative disorder caused by aberrant wound healing following skin injuries such as burns, lacerations, and surgery, is characterized by invasive growth of fibroblasts and excessive Extracellular Matrix (ECM) accumulation.^1^ The destruction of skin tissue structure may lead to various degrees of dysfunction of tissues or organs, and even disability, making HS become an increasing social health problem.^2^ According to statistics, the incidence of scars after surgery in Chinese is 70%, and the incidence of scars in burn patients is as high as 90%.^3^ Currently, the therapy of HS includes surgical resection, laser treatment, and silicone gel sheeting.^4^ However, due to individual differences, the outcomes remain unsatisfactory.^5^ Therefore, it is imperative to identify the molecular mechanisms underlying HS formation for developing novel therapeutic strategies for HS.

Autophagy is a pivotal process for the turnover of intracellular substances in eukaryotes, during which the damaged organelles or proteins are wrapped by autophagic vesicles, and then sent to vacuoles or lysosomes for recycling and degradation.^6^ Autophagy is a cellular response to maintain the homeostasis of tissue structure and function.^7^ Autophagy plays a crucial role in the pathogenesis of numerous diseases.^8^ Furthermore, autophagy was reported to be tightly associated with the survival, differentiation, and maintenance of fibroblasts during the wound healing process, which indicates that the dysregulation of autophagy is a pathological basis of HS formation.^9^, ^10^

Angiopoietin-2 (ANGPT2), as a growth factor, belongs to the angiopoietin/Tie pathway.^11^ It is a 496 amino acid-long protein with a COOH-terminal fibrinogen-like domain, an NH2-terminal coiled-coil domain, and a secretion signal peptide.^12^ ANGPT2 was identified as a significant mediator of renal fibrosis and autophagy in diabetic nephropathy, whose knockdown increased autophagy level and attenuated renal fibrosis via activating the MEK/ERK/Nrf-1 pathway.^13^ In the previous study, ANGPT2 was found to be overexpressed (fold change > 20) in fibroblasts derived from HS tissues compared with fibroblasts from normal skin tissues.^14^ However, whether ANGPT2 participates in autophagy during HS formation and the potential molecular mechanisms are not clear. Furthermore, ANGPT2 was demonstrated to regulate the Phosphoinositide 3-Kinase (PI3K)/Akt signaling pathway.^15^ The PI3K/Akt pathway is a major upstream modulator of the kinase mammalian Target of Rapamycin (mTOR), which is a complex of two mTOR components, mTORC1 and mTORC2, and mTORC1 is a major negative regulator of autophagy.^16^ Therefore, the PI3K/Akt/mTOR pathway has been identified to be closely associated with autophagy during the development of many human diseases.^17^ The present study is intended to figure out whether ANGPT2 regulates autophagy during HS formation via the PI3K/Ak/mTOR pathway.

In the present study, the authors first detected ANGPT2 expression in HS tissues and Hypertrophic Scar Fibroblasts (HSFs) compared to normal tissues and normal skin fibroblasts. HSF autophagy, growth, migration, and ECM accumulation were evaluated after ANGPT2 downregulation to investigate the role of ANGPT2 in HS formation, and the detailed regulatory mechanism was also explored subsequently. The findings in the present study might provide a new potential therapeutic target for HS.

## Materials and methods

### Tissue collection

HS tissues at 1, 3, 6 and 12 months after deep second-third degree burn and adjacent normal skin tissues were obtained from 40 adult patients who were diagnosed with HS by routine pathological examinations and had undergone surgical excision at the General Hospital of Central Theater Command of People’s Liberation Army. All patients (age range, 23‒59 years old; gender ratio, 1:1) were Asian with yellow skin color. Among all 40 cases, there were 16 cases of hydrothermal scald, 13 cases of flame burn, 8 cases of steam scald, and 3 cases of electric injury. The cases involved various body parts, including the upper limbs (n = 15), lower limbs (n = 12), chest (n = 8), and abdomen (n = 5). Before the excision of HS tissues, the skin was sanitized with 3% hydrogen peroxide and then depilated using a disposable razor (BIC® Twin Lady Sensitive, Société BIC, France) with shaving foam. During the surgery, HS tissues were completely excised, and normal skin tissues were obtained after trimming. All tissues were immediately frozen in liquid nitrogen and stored at −80 °C for later preparation of total RNA and total protein lysates.

Included in this study were patients with HS identified by clinicians. No patients received any local laser, drug, or other scar treatment as well as any hormonal medication in 3 months prior to the surgery. Patients with any other infectious, immune, or skin diseases were excluded. Before surgery, all patients were informed of the purpose and procedures of the study and agreed to offer their excised tissues. Written informed consent was obtained from all participators. All protocols in this study were approved by the Ethics Committee of the General Hospital of Central Theater Command of People’s Liberation Army.

### Cell culture

HSFs and normal fibroblasts purchased from the American Type Culture Collection (ATCC, Manassas, VA, USA) were incubated in Dulbecco's modified Eagle's medium (DMEM, Gibco, Carlsbad, CA, USA) containing 10% fetal bovine serum (FBS, Gibco). All cells were maintained in a humidified atmosphere with 5% CO_2_ at 37 °C.

### Cell transfection

HSFs were seeded into 6-well plates and incubated at 37 °C until 80% confluence prior to cell transfection. The short hairpin RNA (shRNA) targeted against ANGPT2 (sh-ANGPT2) and a scrambled shRNA as Negative Control (sh-NC) were synthesized by GenePharma (Shanghai, China). Then, they were transfected into HSFs using Lipofectamine 3000 (Invitrogen). Twenty-four h post-transfection, transfection efficiency was evaluated by RT-qPCR.

### Reverse transcription quantitative polymerase chain reaction (RT-qPCR)

Total RNA was extracted from HS tissues and HSFs using TRIzol reagent (Invitrogen). In brief, 500 ng of total RNA was reverse transcribed into cDNA using a cDNA Reverse Transcription Kit (Takara, Japan). The cDNA amplification was carried out with an SYBR Green PCR kit (Takara) on the ABI 7500 system (Applied Biosystems, Foster, CA). GAPDH served as an internal reference to normalize the expression of target genes. Target gene expression levels were quantified using the 2^-ΔΔCt^ method. Primer sequences for PCR were as follows: GAPDH forward, 5′-TCATTTCCTGGTATGACAACGA-3′ and reverse, 5′-GTCTTACTCCTTGGAGGCC-3′; ANGPT2 forward, 5′-CAATTATTCAGCGACGTGAG-3′ and reverse, 5′-AAGGGTTACCAAATCCCAC-3′.

### Western blotting

The protein levels of ANGPT2 in HS tissues and HSFs as well as the protein levels of PI3K/AKT/mTOR pathway markers (PI3K, phospho-PI3K [p-PI3K], Akt, phospho-Akt [p-Akt], mTOR, phospho-mTOR [p-Mtor]), cell migration markers (Matrix Metalloproteinases 2 [MMP2, MMP9]), ECM markers (alpha-smooth muscle actin [α-SMA], Collagen type 1 [Col 1 and Col 3]) and autophagy markers (LC3B, P62, Beclin-1 and autophagy-related 5 [ATG5]) in HSFs were measured with western blotting. Total protein was extracted from HS tissues or HSFs using radioimmunoprecipitation assay buffer (Beyotime) containing protease inhibitors (Beyotime). Protein concentration was determined using a bicinchoninic acid protein assay kit (Beyotime). Then, equal amounts of extracted proteins were subjected to Sodium Dodecyl Sulfate-Polyacrylamide Gel Electrophoresis (SDS-PAGE) and then transferred onto a PVDF membrane (Millipore, Bedford, MA, USA). The membrane was blocked with 5% skimmed milk for 1 h, followed by incubation with primary antibody against ANGPT2 (1:1000, ab199133), PI3K (1:1000, ab191606), p-PI3K (1:1000, ab138364), Akt (1:2000, ab32505), p-Akt (1:1000, ab38449), mTOR (1:10000, ab134903), p-mTOR (1:1000, ab109268), MMP2 (1:1000, ab92536), MMP9 (1:1000, ab76003), Col 1 (1:1000, ab275746), Col 3 (1:5000, ab7778), α-SMA (1:10000, ab124964), LC3B (1:2000, ab192890), P62 (1:1000, ab155686), Beclin-1 (1:2000, ab207612), ATG5 (1:1000, ab108327) and GAPDH (1:10000, ab181602) at 4 °C overnight, all bought from Abcam (Cambridge, UK). On the next day, the membrane was rinsed thrice with TBST and then incubated with HRP-conjugated secondary antibodies at room temperature for 2 h. The protein bands were detected using an ECL detection kit (Thermo Fisher Scientific).

### Cell Counting Kit-8 (CCK-8) assay

CCK-8 assay was employed for assessing cell proliferation ability. After 48 h transfection with sh-NC or sh-ANGPT2, HSFs were seeded into 96 well plates (5 × 10^3^ cells/well). Twenty-four h later, 10 µL of CCK-8 solution (Sigma-Aldrich) was added to each well. After 2 h of incubation, the proliferation of HSFs was assessed by measuring the absorbance at different time points (24, 48 and 72 h) using a microplate reader at 450 nm.

### Transwell migration assay

The Transwell chamber (8 μm diameter, Corning) was used to measure cell migratory ability. The lower chamber was filled with 500 μL of 20% FBS medium. After 48 h of transfection, HSFs were harvested, washed with PBS, and resuspended in DMEM without FBS. Then, 200 μL of cell suspension (1 × 10^5^ cells) were loaded onto the upper chamber and then cultured for 24 h at 37 °C. The nonmigratory cells were gently removed with a cotton swab. The migratory cells were stained with 0.5% crystal violet after fixation with 100% methanol and visualized under an inverted microscope.

### Statistical analysis

Data of at least three independent experiments are expressed as the mean ± standard deviation. The SPSS 20.0 software (IBM, Chicago, IL, USA) was used for statistical analysis. Statistical analysis of the data using the Shapiro-Wilk normality test showed that all data followed a normal distribution. Therefore, statistical comparisons containing two groups or more groups were estimated by Student’s *t*-test or one-way analysis of variance (ANOVA) followed by Tukey's *post-hoc* test. A value of p < 0.05 was considered to denote a statistically significant difference.

## Results

### ANGPT2 is upregulated in HS tissues and HSFs

**Figure 1:**
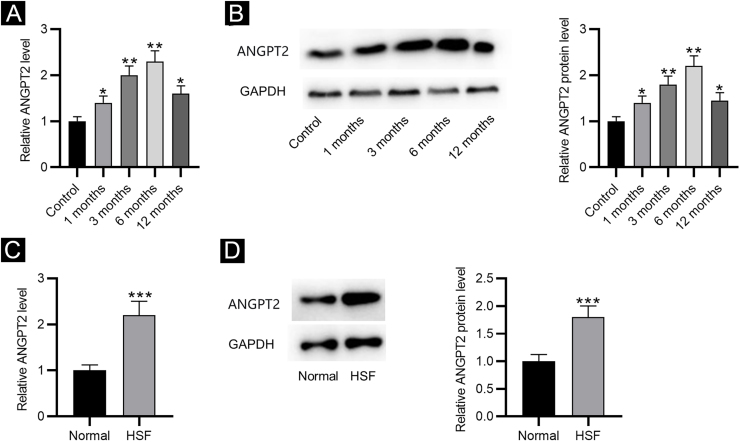
**The expression of ANGPT2 in HS tissues and HSFs.** (A–B) ANGPT2 expression in HS tissues versus normal skin tissues were assessed by RT-qPCR and western blotting. (C–D) ANGPT2 expression in HSFs compared to normal fibroblasts was determined by RT-qPCR and western blotting. *p < 0.05, **p < 0.01, ***p < 0.001.

First, the results of RT-qPCR and western blotting revealed that the expression and protein level of ANGPT2 were gradually elevated in HS tissues at 1, 3 and 6 months after burning, and were partially recovered at 12 months (Fig. 1A‒B). Furthermore, ANGPT2 also presented notably upregulated expression and protein level in HSFs versus normal fibroblasts (Fig. 1C‒D). Therefore, ANGPT2 is upregulated in HS tissues and HSFs.

### Inhibition of ANGPT2 inactivates the PI3K/Akt/mTOR pathway in HSFs

**Figure 2:**
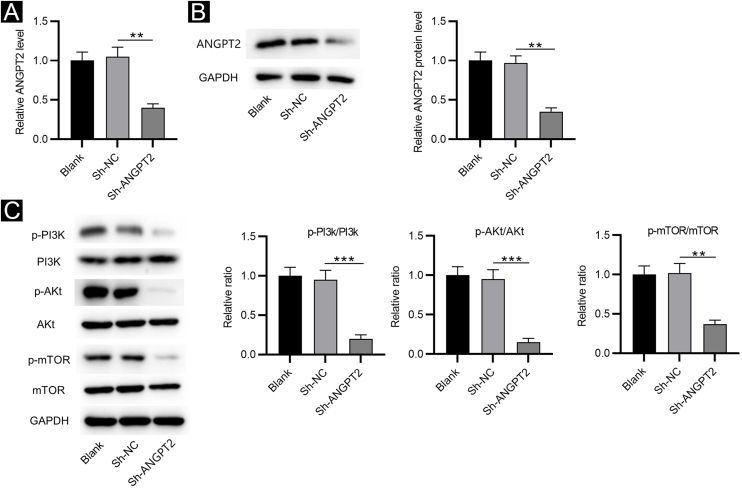
**The effects of ANGPT2 knockdown on the PI3K/Akt/mTOR pathway in HSFs.** (A‒B) The expression of ANGPT2 in HSFs transfected with sh-ANGPT2 or sh-NC was measured by RT-qPCR and western blotting. (C) The levels of PI3K/Akt/mTOR pathway-related proteins in HSFs after ANGPT2 downregulation were measured by western blotting. **p < 0.01, ***p < 0.001.

RT-qPCR and western blotting showed that ANGPT2 expression was significantly reduced in HSFs transfected with sh-ANGPT2 versus sh-NC group (Fig. 2A‒B). ANGPT2 knockdown dramatically reduced the protein levels of p-PI3K, p-Akt and p-mTOR in HSFs versus the sh-NC group (Fig. 2C). These data indicated that ANGPT2 downregulation inactivates the PI3K/Akt/mTOR pathway in HSFs.

### Inhibition of ANGPT2 activates autophagy via suppressing the PI3K/Akt/mTOR pathway

**Figure 3:**
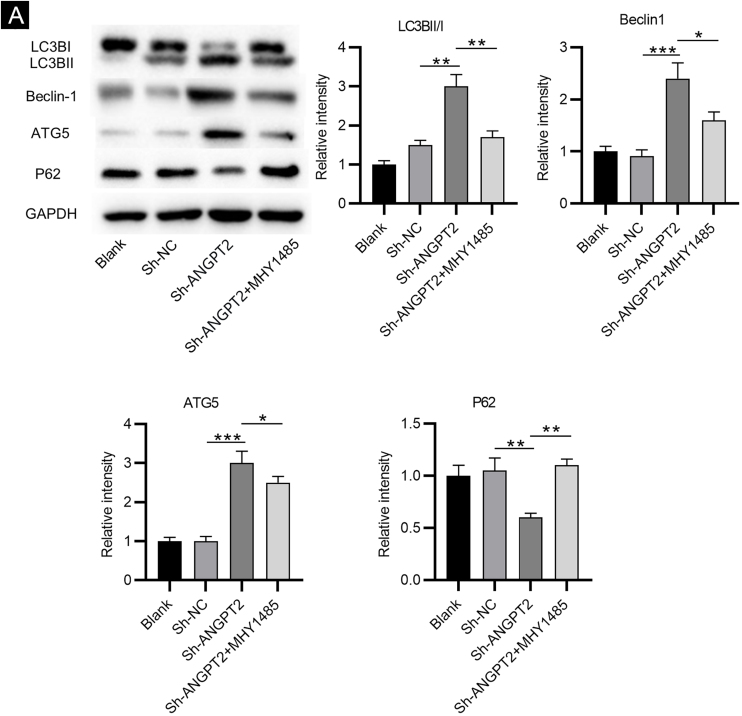
**The effects of ANGPT2 knockdown on the autophagy in HSFs.** (A) Western blotting was performed to evaluate levels of autophagy-related LC3B Ⅰ, LC3B ⅠⅠ, Beclin-1, ATG5 and P62 proteins in HSFs transfected with sh-NC, sh-ANGPT2, or sh-ANGPT2+MHY1485. *p < 0.05, **p < 0.01, ***p < 0.001.

As shown by western blotting, the protein levels of LC3B Ⅰ and P62 were markedly reduced, while the protein levels of LC3B II, Beclin-1 and ATG5 were remarkably elevated in HSFs after downregulating ANGPT2. The ratio of LC3B II and LC3B I was found to be obviously increased after ANGPT2 knockdown (Fig. 3A). In addition, the authors also that the treatment of MHY1485, an mTOR activator, reversed the influence of ANGPT2 downregulation on the protein levels of the above autophagy markers in HSFs (Fig. 3A‒E). Therefore, the authors concluded that inhibition of ANGPT2 activates autophagy through repressing the PI3K/Akt/mTOR pathway in HSFs.

### Inhibition of ANGPT2 improves cell dysfunction by suppressing the PI3K/Akt/mTOR pathway

**Figure 4:**
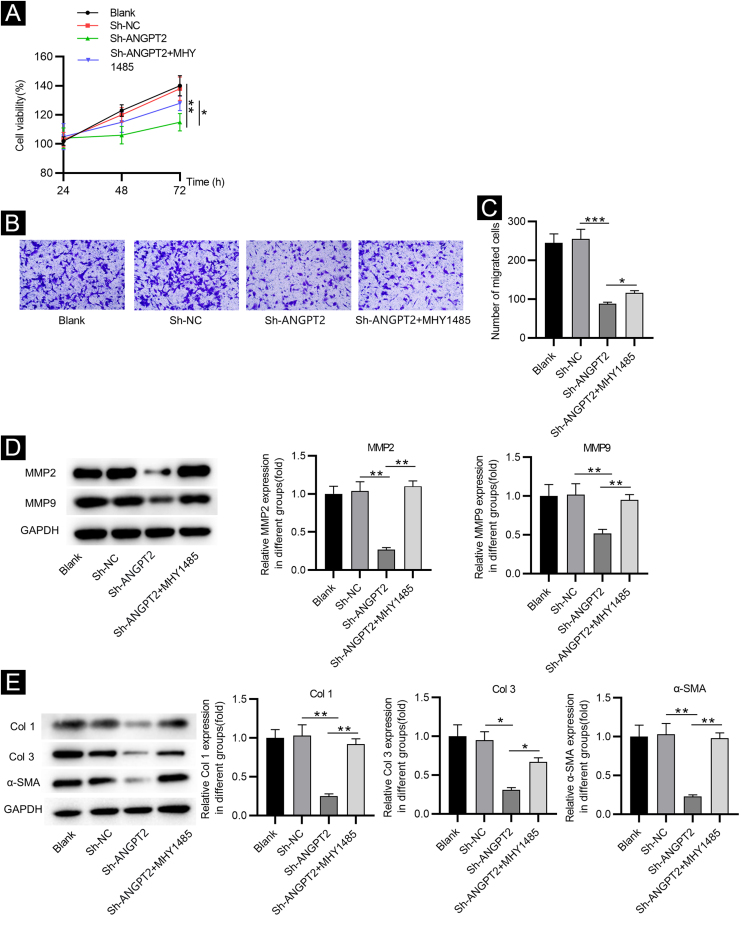
**The effects of ANGPT2 knockdown on the proliferation and migration of HSFs as well as ECM accumulation.** (A) CCK-8 assay was conducted to examine the proliferation of HSFs in Blank, sh-NC, sh-ANGPT2, and sh-ANGPT2+MHY1485 groups. (B‒C) Transwell assay was applied to assess the migration of HSFs in the above four groups. (D) Levels of migration-related proteins in the above four groups were measured by western blotting. (E) Western blotting was carried out to evaluate the expression of ECM-related proteins in the above four groups. *p < 0.05, **p < 0.01, ***p < 0.001.

CCK-8 assay demonstrated that the proliferation of HSFs was inhibited after ANGPT2 downregulation, while MHY1485 treatment partially reversed this change (Fig. 4A). ANGPT2 knockdown also repressed the migration of HSFs, which was partially restored after MHY1485 treatment (Fig. 4B‒C). The levels of MMP2 and MMP9 were reduced after ANGPT2 downregulation, which was restored following the subsequent MHY1485 treatment (Fig. 4D). Additionally, a significant decrease of α-SMA, Col 1 and Col 3 protein levels was observed in HSFs transfected with sh-ANGPT2. However, the addition of MHY1485 attenuated the inhibitory influence of ANGPT2 knockdown on α-SMA, Col 1 and Col 3 levels (Fig. 4E). In conclusion, ANGPT2 knockdown alleviates abnormal proliferation, migration, and ECM accumulation in HSFs during HS formation via suppressing the PI3K/Akt/mTOR pathway.

## Discussion

HS is a prevalent complication during the wound healing process, which brings great pain to patients.^18^ The current therapy for HS including surgical resection, laser therapy, pressure garment therapy, and the use of anti-scar drugs such as silicone gel usually has limitations, and the outcome is not so satisfactory.^19^ Therefore, the present study intends to explore the molecular mechanisms underlying the process of HS formation, thus discovering potential targets for HS treatment. In this study, the authors discovered that ANGPT2, which was upregulated in HS tissue and HSFs, activated autophagy via inhibiting the PI3K/Akt/mTOR pathway. In addition, ANGPT2 knockdown attenuated the excessive growth, migration, and ECM accumulation by inhibiting the PI3K/Akt/mTOR pathway, which alleviated HS formation.

HS formation is mainly caused by aberrant proliferation and migration of HSFs as well as excessive ECM deposition.^20^ Previously, many studies have reported that HS formation could be alleviated by suppressing the abnormal growth of HSFs and excessive deposition of ECM. For example, miR-519d reduces the proliferation and induces the apoptosis of HSFs, and represses the expression of ECM-related genes, thus inhibiting HS formation.^21^ p75NTR downregulation suppresses growth, migration, and ECM accumulation of HSFs, thus alleviating HS formation.^22^ ANGPT2 is a secreted protein and a member of the angiopoietin family, being a representative key factor for angiogenesis and vascular remodeling.^23^ Angiogenesis plays a critical role in the wound-healing process, and reports demonstrate that hypertrophic scars contain more microvessels than the normal dermis.^24^ So far, anti-angiogenic therapy has been confirmed as an effective strategy for early intervention of HS and has been used to alleviate HS formation.^25^ ANGPT2 was found to be significantly overexpressed in fibroblasts derived from HS tissues compared with fibroblasts from normal skin tissues in the previous study,^14^ from which the authors inferred that might participate in the process of HS formation. In the present study, the authors first found that ANGPT2 expression was higher in HS tissues and HSFs than in normal skin tissues and normal skin fibroblasts. Then the authors downregulated ANGPT2 in HSFs, where it was discovered that the abnormal proliferation and migration were suppressed. MMPs were reported to play significant roles in various stages of the wound healing process, among which MMP2 and MMP9 are closely related to the migration of fibroblasts.^26^ Through western blotting, the authors also discovered that MMP2 and MMP9 levels in HSFs were reduced after ANGPT2 knockdown. Additionally, as we know, excessive deposition of ECM will result in the fibrosis of organs, including the skin, the largest organ of the human body.^27^ Collagen is the most important component of the ECM.^28^ The expression of α-SMA has also been shown to increase as the degree of organ fibrosis increases.^29^ Col 1, Col 3, and α-SMA were demonstrated to be visibly enhanced in HS, and function vitally during the formation of a fibrotic ECM environment.^30^ Then, the authors detected the levels of α-SMA, Col 1, and Col 3 in HSFs after downregulating ANGPT2 using western blotting, which indicated that ANGPT2 knockdown reduced the protein levels of α-SMA, Col 1, and Col 3. Therefore, inhibition of ANGPT2 was verified to suppress the growth, migration, and ECM accumulation in HSFs, which alleviates the development of HS.

Autophagy refers to the process that the double-layer membrane shed from the ribosome attachment zone of the rough endoplasmic reticulum wraps part of the cytoplasm and the organelles and proteins that need degradation in the cell to form autophagosomes.^31^ As a tightly modulated physiological process, autophagy is important for cellular development, differentiation, and maintenance.^32^ The activation of autophagy was reported to induce the suppression of cell proliferation, migration, and ECM accumulation.^33^, ^34^ In the previous study, the reduced autophagic capacity of fibroblasts was associated with the pathogenesis of HS.^35^ Then, the authors also investigated the influence of ANGPT2 knockdown on HSF autophagy. ATG5, a structural protein made up of an α-Helical Bundle Region (HBR) and two Ubiquitin-like-Fold Domains (UFDs), is part of the ATG12-ATG5 complex involved in autophagosome formation or elongation, acting as an E3-like enzyme in LC3 lipidation.^36^ During the autophagy process, LC3 Ⅰ is coupled with Phosphatidylethanolamine (PE) in the presence of ATG5/ATG12 to form LC3 ⅠⅠ, which is located on the inner and outer membranes of autophagy.^37^ The presence of LC3 II is considered a symbol of autophagosome formation.^38^ P62 is a multifunctional adaptor protein that is usually selected as the substrate of autophagy.^39^ In intact autophagy, p62 has a short LC3 interaction region that promotes direct interaction with LC3 and causes p62 to be specifically degraded by autophagy.^40^ The level of p62 has been used as a marker for inhibition of autophagy or defects in autophagic degradation.^41^ Beclin-1, as a mature core mammalian autophagy protein, is also an important factor in the regulation of autophagy.^42^ In previous studies, the change in the levels of LC3B I, LC3B II, p62, ATG5, and Beclin-1 was evaluated to investigate autophagy.^43^, ^44^, ^45^ In the present study, the levels of these autophagy-associated proteins in HSFs were also examined after ANGPT2 downregulation. The authors discovered that ANGPT2 knockdown reduced the levels of LC3B Ⅰ and P62, and increased the levels of LC3B II, ATG5, and Beclin-1, which demonstrated the activation of autophagy. Therefore, the authors concluded that the inhibition of ANGPT2 on HSF growth, migration, and ECM accumulation was achieved by autophagy activation.

Many pathways are involved in autophagy, among which the PI3K/Akt/mTOR pathway has been widely investigated.^46^ It was reported in previous studies that during the development of multiple diseases, cell autophagy is activated via suppressing the PI3K/Akt/mTOR pathway. For example, PSORI-CM02 induces autophagy by inhibiting the PI3K/Akt/mTOR pathway, thereby alleviating the development of psoriasis, an inflammatory skin disease.^47^ Exogenous hydrogen sulfide restrains human skin melanoma development by facilitating autophagy in melanoma cells via the repression of the PI3K/AKT/ mTOR pathway.^48^ Furthermore, during HS formation, p75NTR silencing was reported to activate autophagy by inhibiting the PI3K/Akt/mTOR pathway.^22^ Therefore, the authors explored whether ANGPT2 knockdown exerts effects on autophagy during the process of HS formation via mediating the PI3K/Akt/mTOR pathway. The authors initially detected the protein levels of p-PI3K, p-Akt, and p-mTOR in HSFs, which showed a significant reduction after ANGPT downregulation. This suggested that the PI3K/Akt/mTOR pathway was restrained by ANGPT downregulation. Subsequently, to confirm the regulatory mechanism of ANGPT2, the mTOR agonist MHY1485 was used to activate this pathway. The authors observed that MHY1485 treatment reduced the activity of autophagy in ANGPT2-downregulated HSFs versus cells with ANGPT2 downregulation alone. Similarly, the inhibition of ANGPT2 downregulation on growth, migration, and ECM accumulation of HSFs were partially restored after MHY1485 treatment. Collectively, ANGPT2 downregulation repressed growth, migration, and ECM accumulation of HSFs via autophagy activation by inhibiting the PI3K/Akt/mTOR pathway.

## Conclusion

The present study demonstrated for the first time that inhibition of ANGPT2 activated autophagy via suppressing the PI3K/Akt/mTOR pathway. Additionally, ANGPT2 knockdown further inhibited the proliferation, migration, and ECM accumulation in HSFs through the above mechanism. These results indicate that ANGPT2 might act as a novel potential target to alleviate HS formation.

## Financial support

This work was supported by the Natural Science Foundation of Hubei Province ( 2020CFB210).

## Authors' contributions

Hongxin Chen: Study conception and planning; collection, analysis, and interpretation of data; Statistical analysis; drafting and editing of the manuscript; Critical review of the manuscript; Approval of the final version of the manuscript.

Kai Xu: Study conception and planning; collection, analysis, and interpretation of data; Statistical analysis; Drafting and editing of the manuscript; Critical review of the manuscript; Approval of the final version of the manuscript.

Chao Sun: Collection, analysis, and interpretation of data; Critical review of the literature; Approval of the final version of the manuscript.

Si Gui: Collection, analysis, and interpretation of data; Critical review of the literature; Approval of the final version of the manuscript.

Juanjuan Wu: Collection, analysis, and interpretation of data; Critical review of the literature; Approval of the final version of the manuscript.

Song Wang: Effective participation in research orientation; Collection, analysis, and interpretation of data; Statistical analysis; Preparation and writing of the manuscript; Critical review of the manuscript; Approval of the final version of the manuscript.

## Conflicts of interest

None declared.
